# The Potential Benefits of Applying Recent Advances in Esophageal Motility Testing in Patients with Esophageal Atresia

**DOI:** 10.3389/fped.2017.00137

**Published:** 2017-06-21

**Authors:** Nathalie Rommel, Maissa Rayyan, Charlotte Scheerens, Taher Omari

**Affiliations:** ^1^Neurogastroenterology and Motility, Gastroenterology, University Hospitals Leuven, Leuven, Belgium; ^2^Experimental Otorhinolaryngology, Department of Neurosciences, Deglutology, University of Leuven, Leuven, Belgium; ^3^Neonatal Intensive Care Unit, University Hospitals Leuven, Leuven, Belgium; ^4^Department of Development and Regeneration, University of Leuven, Leuven, Belgium; ^5^School of Medicine, Flinders University, Adelaide, SA, Australia

**Keywords:** esophageal atresia, dysphagia, dysmotility, high-resolution manometry, pressure flow analysis

## Abstract

Infants and children with esophageal atresia commonly present with swallowing dysfunction or dysphagia. Dysphagia can lead to a range of significant consequences such as aspiration pneumonia, malnutrition, dehydration, and food impaction. To improve oral intake, the clinical diagnosis of dysphagia in patients with esophageal atresia should focus on both the pharynx and the esophagus. To characterize the complex interactions of bolus flow and motor function between mouth, pharynx, and esophagus, a detailed understanding of normal and abnormal deglutition is required through the use of adequate and objective assessment techniques. As clinical symptoms do not correlate well with conventional assessment methods of motor function such as radiology or manometry but do correlate with bolus flow, the current state-of-the-art diagnosis involves high-resolution manometry combined with impedance measurements to characterize the interplay between esophageal motor function and bolus clearance. Using a novel pressure flow analysis (PFA) method as an integrated analysis method of manometric and impedance measurements, differentiation of patients with impaired esophago-gastric junction relaxation from patients with bolus outflow disorders is clinically relevant. In this, pressure flow matrix categorizing the quantitative PFA measures may be used to make rational therapeutic decisions in patients with esophageal atresia. Through more advanced diagnostics, improved understanding of pathophysiology may improve our patient care by directly targeting the failed biomechanics of both the pharynx and the esophagus.

## Introduction

In EA, the resulting congenital malformation causes disruption to neural pathways and luminal continuity; further, the required esophageal repair via creation of surgical anastomosis may alter luminal compliance, and together, these factors lead to dysphagia and potentially life-threatening bolus hold up. Diagnostic investigations for esophageal dysphagia aim to describe esophageal anatomy and peristaltic function. Radiological upper gastrointestinal studies can visualize structural abnormalities in the esophagus, such as strictures; however, the motility of the esophagus that arises through CNS and ENS mechanisms is best elucidated using high-resolution manometry (HRM), ideally combined with impedance topography.

## Current Diagnostic Methods to Investigate Dysphagia in EA

**Figure 1:**
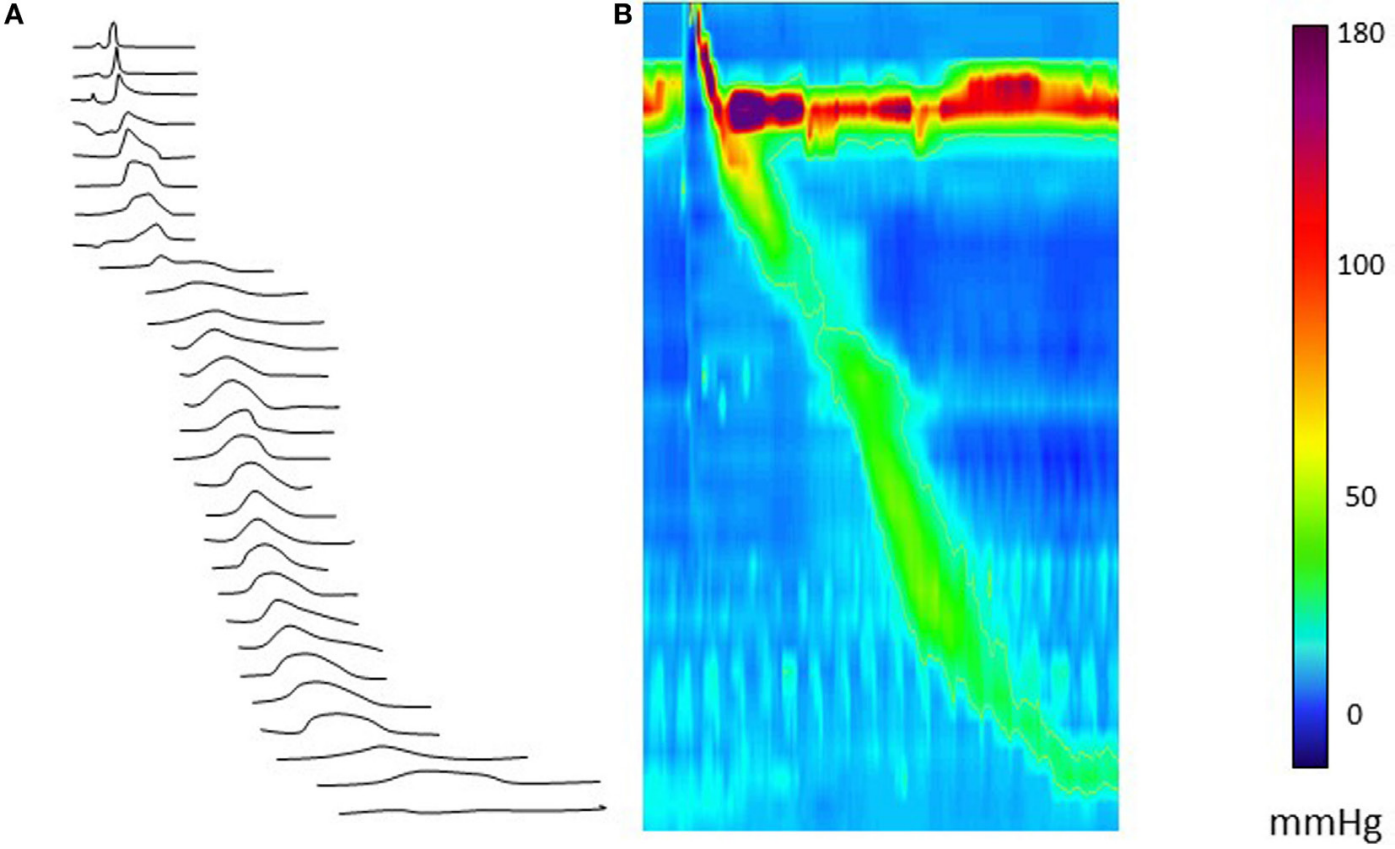
Esophageal high-resolution manometry tracing of a normal liquid swallow, presented as a line plot **(A)** and as a color (Clouse) plot line plot **(B)**. The color panel shows the corresponding pressure values.

In EA patients, an esophageal anastomotic stricture index was proposed to diagnose esophageal strictures (1). Although esophageal function is often clinically assessed using radiological esophagram, manometry has been the diagnostic tool of choice to evaluate esophageal motor function. Through the innovation of HRM, the clinical applicability of esophageal manometry has been revolutionized by improved reliability of the equipment, increased resolution of sensors, the change from perfused to solid state measurements, and the decreased catheter diameter (2). For children with EA, the catheter technology has been suitably miniaturized improving procedural tolerance. HRM is worldwide accepted as a diagnostic tool that offers new perspectives to identify motility patterns through visualization of pressure patterns, as line tracings as well as pressure topography color plots (also known as “Clouse” plots) (Figure 1). Based on these plots, different patterns of motor function can be plotted, recognized, and categorized into a diagnostic algorithm called “the Chicago Classification” (CC), providing normative values and guidelines for evaluating esophageal motor function (3). The CC differentiates four categories of esophageal motor dysfunction: (1) disorders of esophago-gastric junction (EGJ) outflow obstruction (including achalasia); (2) major disorders of peristalsis (including distal esophageal spasm, jackhammer esophagus, and absent contractility); (3) minor disorders of peristalsis (including ineffective motility and fragmented peristalsis); and (4) normal motor function. When using the CC in pediatrics, adjustments for age and size cutoffs are needed as shorter esophageal length and smaller esophago-gastric function diameter influence the metrics (3). Therefore, the available diagnostic criteria need to be adjusted for age and size, specifically the integrated relaxation pressure (IRP4) reflecting deglutitive EGJ relaxation and distal latency (3). Although the CC appears to be applicable for use in the general pediatric population (4, 5), its use in EA as a specific patient subgroup requires further consideration. EA patients often show no motor patterns, and therefore bolus transport to, and through, the EGJ needs to be considered. The pattern of bolus transport and esophageal emptying into the stomach is important to elucidate.

In clinical practice, the interpretation of the HRM motor patterns alone does not easily elucidate aberrant bolus flow, which may lead to symptom generation. Therefore, the evaluation of pressure in relation to bolus flow as measured by manometry with impedance monitoring (a technique with a long-standing history of use in both adult and pediatric populations) has been suggested as a method to also assess esophageal function in children with EA. Combining these diagnostic tools allows assessment of the interplay between structural and functional capacity of the esophagus. Although manometry and impedance can be easily acquired simultaneously, the currently applied paradigm of independent analysis of both recordings has largely failed to bring the anticipated diagnostic gain and to determine a relation with clinical symptoms (6, 7). A lack of sensitivity of the used technologies and/or the absence of an integrated analysis method of manometry and impedance recordings or the fact that normal clearance can also be achieved with abnormal motility patterns may be potential reasons (8). Given children with EA may undergo many radiological investigations over their lifetime, a non-radiological alternative for radiology requires investigation (9–11).

## Pressure Flow Analysis (PFA) to Investigate Dysphagia

Over the last 5 years, the methodology for combined pressure-impedance analysis has developed to the point where it allows for objective, integrated analysis of simultaneously recorded esophageal motility (from pressure topography) and bolus flow (from impedance topography) (5, 12, 13). It is hoped that this method can provide additional physiological and pathophysiological insights because the impedance segments enhance the assessment of bolus flow and clearance/bolus residual. Further, when combined with pressure, impedance can be used to map the point of maximal luminal distension, pinpointing exactly where intrabolus distension pressure (IBP) should be optimally derived. Esophageal symptoms due to a motility disorder generally occur as a response to increased esophageal wall tension because of bolus retention and/or increased IBP, and our ability to directly measure these features therefore enhances the evaluation of esophageal symptoms. Hopefully, this can better guide the approach to diagnosis and management of esophageal disease through objective longitudinal measurements before and after medical/surgical intervention. These newer approaches of combining and analyzing pressure and impedance measurements are collectively called “pressure flow analysis.” PFA was first validated for pharyngeal dysphagia in adults (14, 15) and subsequently has been applied for the evaluation of esophageal dysphagia (12).

A number of studies support the notion that the pressure flow approach can better detect flow resistance and esophageal stasis in patients with dysphagia (16, 17). More recently, new pressure flow measures have been found to reliably detect flow-permissive conditions that predict bolus emptying across the EGJ (18–20). Furthermore, while seemingly complex, derivation of pressure flow measures is relatively easy to apply using software that only requires the analyst to identify space-time landmarks on the pressure map of a swallow. Such software has been found to be reliable in the hands of analysts with differing levels of expertise (21).

**Figure 2:**
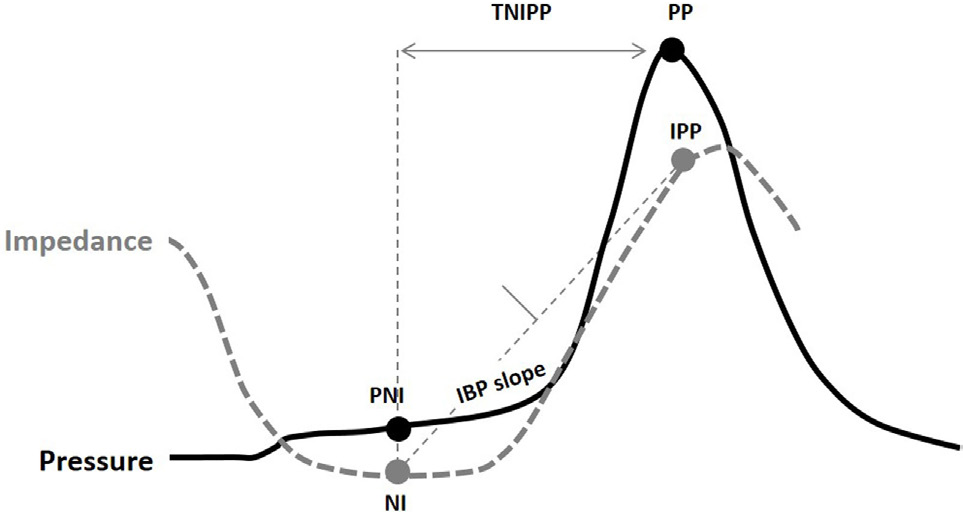
Pressure flow analysis metrics indicated on a combined pressure (black) and impedance (gray) line plot [Omari et al. (24)]. Abbreviations stand for NI, nadir impedance; PP, peak pressure; IPP, impedance at peak pressure; PNI, pressure at nadir impedance; IBP-slope, intrabolus pressure slope; TNIPP, time from nadir impedance to peak pressure.

Some of the key PFA metrics currently being evaluated are described in Table 1 and illustrated in Figure 2. Some studies suggest utility for the evaluation of dysphagia (16, 17, 22). Further, a composite score based on three key variables, called the pressure flow index (PFI), has been derived. The PFI quantifies bolus pressurization relative to flow. A second global measure, called the impedance ratio (IR), quantifies bolus retention. A further extension of the PFA paradigm is to plot swallows on a “pressure flow matrix” (13, 16); this matrix visually depicts the PFI with the IR, allowing dichotomous separation of swallows associated with abnormal bolus clearance (vertical axis) and/or those associated with abnormal bolus flow resistance (horizontal axis) (16, 23).

**Table 1:** Pressure flow metrics.

Nadir impedance	NI	Ohms	Bolus presence
Peak pressure	PP	mmHg	Pressure recorded at maximum contractile tension
Impedance at peak pressure	IPP	Ohms	Bolus presence at time of maximum contractile tension
Impedance ratio: nadir impedance to impedance at peak pressure ratio	IR		Marker for incomplete bolus transit
Pressure at nadir impedance	PNI	mmHg	Intrabolus pressure (IBP) recorded when the esophageal lumen is maximally filled by the bolus
Intrabolus pressure	IBP	mmHg	IBP recorded during luminal emptying
Intrabolus pressure slope	IBP-slope	mmHg	Rate of change in IBP recorded during luminal emptying
Time from nadir impedance to peak pressure	TNIPP	s	Time interval from maximally full lumen to maximal contractile tension
Pressure flow index	PFI (IBP × distal IBP-slope)/(TNIPP) ratio		Relationship between peristaltic strength and flow resistance in the distal esophagus

**Figure 3:**
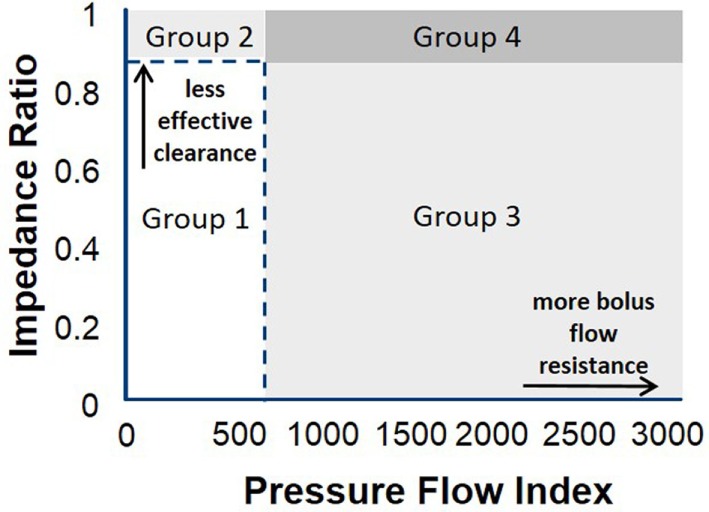
Pressure flow matrix: this matrix visually presents the combination of pressure flow index (PFI) with the impedance ratio (IR), aiming to dichotomously separate outpatients with dysphagia who have predominantly abnormal bolus clearance and/or those with abnormal bolus resistance at the esophago-gastric junction (EGJ) (16). The pressure flow matrix shows on the vertical axis the bolus data of patients with normal and abnormal flow resistance and on the horizontal axis the bolus data of patients with normal and abnormal bolus clearance. Depending on the combined value of these two metrics, the predominant pressure flow pattern becomes clear. The matrix consists of four quadrants and indicate the following groups: Group 1: patients with normal effective transit and normal flow resistance across the EGJ; Group 2: ineffective transit and normal bolus flow resistance across the EGJ; Group 3: effective transit but increased bolus flow resistance across the EGJ; Group 4: ineffective transit and increased bolus flow resistance across the EGJ. It is expected that control subjects will have a low PFI and a low IR, and these are indicated by the dotted line. Patients with esophageal atresia are hypothesized to present in Groups 2 and 4, but further research is needed to consolidate this hypothesis (13).

**Figure 4:**
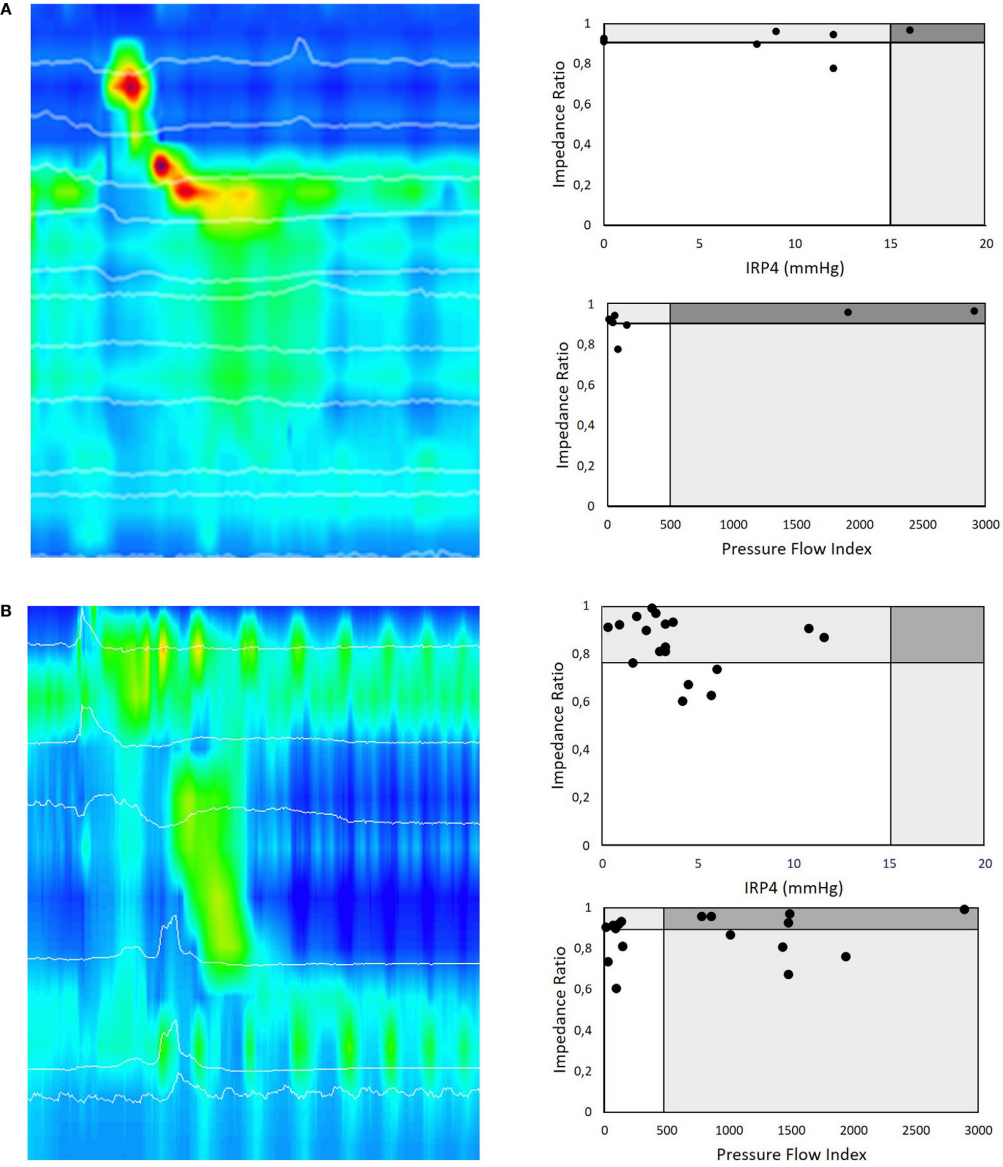
**(A)** HRMI color plot of a liquid swallow in a 16-month-old postoperative patient with Type C esophageal atresia. This girl underwent a primary anastomosis in the neonatal period and nine dilatations for esophageal strictures. Her main complaint was intermittent dysphagia on solids. All liquid swallows of this HRMI study of this patient are presented according to the pressure flow analysis (PFA) matrix paradigm. A first PFA matrix represents the impedance ratio (IR) versus the integrated relaxation pressure (IRP4), a manometric parameter to describe relaxation of the esophago-gastric junction (EGJ) during swallowing. This PFA matrix shows that many of the swallows look normal in terms of deglutitive relaxation as well as bolus clearance. The second PFA matrix of this patient shows the IR versus pressure flow index (PFI) for the same swallows. In this case, the PFA matrix confirms that the (for EA typical pattern) ineffective esophageal motility leads to ineffective esophageal bolus clearance. The EGJ deglutitive relaxation represented by the IRP4 is in most swallows normal and corresponds in this patient with low bolus flow resistance at EGJ as represented by the PFI. This HRMI study also revealed incomplete relaxation of the upper esophageal sphincter that corresponds with recurrent coughing episodes during the examination and her clinical symptoms of dysphagia. **(B)** Similar example of an HRMI color plot of a liquid swallow in a 2-month-old postoperative patient with Type A esophageal atresia. The first PFA matrix shows that many of the swallows have a normal deglutitive relaxation as well as bolus clearance. The second PFA matrix of this patient (IR versus PFI) shows that the PFI is increased in the majority of the swallows and thereby discloses that these swallows are abnormal in terms of bolus transit and clearance. This example illustrates that PFA allows a more differentiating diagnosis than high-resolution manometry assessment alone.

An example of pressure flow matrix data is illustrated in Figure 3. Depending on the combined value of these two metrics across multiple repeat swallows, the predominant pressure flow pattern emerges. Typically healthy control subjects will have a low PFI and a low IR [i.e., will reside in the lower left-hand corner of the matrix (see Figures 3 and 4)]. The other three quadrants of the matrix indicate an abnormal pattern of (a) ineffective transit, (b) increased bolus flow resistance across the EGJ, or (c) ineffective transit and increased bolus flow resistance across the EGJ.

This matrix can be applied to patients with EA. In that case, it can be hypothesized that patients with EA will mainly be classified in Groups 2 and 4 due to the poor clearance capacity of the affected esophagus, but further research is ongoing to confirm this hypothesis and determine if information of this kind is relevant for management of, for example, esophageal anastomotic strictures or in relation to decisions to undertake anti-reflux surgery.

We illustrate this dichotomized PFA approach in clinical practice by presenting two cases (Figure 4). In the first case, we present a 16-month-old girl with Type C esophageal atresia with dysphagia for solids after multiple dilatation for strictures. EPT metrics indicate that the majority of the swallows showed abnormal esophageal peristalsis with complete EGJ function (IRP4 > 15 mmHg) (Figure 4A). In this case, PFA metrics confirm that in the majority of the swallows, the PFI was normal, suggesting no flow resistance during deglutition, as detected by HRM. The PFA matrix shows, however, a highly elevated IR and thereby confirms non-radiologically the inadequate bolus clearance secondary to abnormal contractility and which links in with the patient’s clinical symptoms of dysphagia for solids.

The second example describes a 2-month-old postoperative boy with Type A esophageal atresia with dysphagia. Standard EPT metrics showed abnormal esophageal peristaltic integrity (ICD < 2 cm) and intermittent EGJ function (IRP4s = 3 mmHg) in the majority of the swallows (Figure 4B). However, PFA metrics demonstrated that in the majority of the swallows, the PFI was highly elevated, suggesting high flow resistance during deglutition, not detected by HRM as stand-alone technique. This highly elevated PFI may link to the abnormal bolus flow and thereby correspond with the patient’s symptoms.

### Relevance to the EA Population

In the first year of life, patients with esophageal atresia frequently present with respiratory problems (37%) and also with digestive problems (25). Many patients develop anastomotic stenosis (22–37%), recurrent fistula (4%), gastro-esophageal reflux requiring anti-reflux surgery (12%), or dysphagia (15–52%) (25–27). Throughout life, dysphagia is the most common symptom of patients with EA. Its incidence can vary depending on the definition (25, 26, 28, 29) but seems to be lower in young infants compared to that in children and adults (25–27). Dysphagia is defined as a swallowing disorder in the oral, pharyngeal, and/or esophageal phases of deglutition. Some patients display only mild symptoms and need to drink liquids to facilitate swallowing (30). Other children present with a wider spectrum of symptoms varying from hypersalivation, early satiety, gagging, vomiting, and food refusal (13).

Dysphagia can originate in the oral cavity, pharynx, and esophagus. Typically, patients with EA have normal oral motor function; however, it is important to recognize that oral aversion may be a sign of pharyngeal and/or esophageal dysphagia and is not necessarily directly (causally) linked to abnormal oral responsiveness or sensitivity. Pharyngeal dysphagia, in general, can relate to inadequate pharyngeal motor function and responsiveness, inadequate laryngeal closure, and/or inadequate relaxation and opening of the upper esophageal sphincter (UES). In children with EA, no systematic reports on pharyngeal or UES function are available.

A frequent cause of dysphagia in EA is inadequate motility of the esophagus. Severity is variable and is influenced by the presence of congenital esophageal stenosis and esophageal strictures. At the moment, the most commonly used clinical diagnostic tests to assess esophageal function are the radiological barium study and esophageal manometry. Both methods aim to evaluate the anatomy and motor function of the esophagus and EGJ (6, 30). Dysphagia can be challenging as these traditional methods often fail to explain the clinical symptoms of dysphagia due to poor symptom correlation with the documented esophageal motor patterns. During the last 5 years, PFA became available, an automated analysis method that derives quantitative pressure flow metrics from simultaneously acquired impedance and manometry measurements (24). These pressure flow metrics elucidate the interplay between bolus flow, motor patterns, and symptomatology by combining data on bolus flow resistance and bolus transit. Symptoms of dysphagia and altered perception of bolus passage may indicate increased bolus flow resistance at the EGJ and ineffective esophageal propulsion.

## Conclusion

The clinical diagnosis of dysphagia in patients with esophageal atresia should focus on both the pharynx and the esophagus. As clinical symptoms do not correlate well with conventional assessment methods of motor function such as radiology and manometry but do correlate with bolus flow, the current state-of-the-art diagnosis includes HRM combined with impedance measurements to characterize the interplay between bolus flow and esophageal motor function. Differentiation of patients with impaired EGJ relaxation from patients with bolus outflow disorders is clinically relevant and can be achieved using a novel PFAmethod, which is an integrated analysis method of manometric and impedance measurements; its pressure flow matrix is a useful tool for categorizing the quantitative PFA measures and may be used to make rational therapeutic decisions in patients with esophageal atresia. Through more advanced diagnostics, improved understanding of pathophysiology may improve our patient care by directly targeting the failed biomechanics.

## Author Contributions

Drafting of the manuscript: NR and TO. Critical revision of the manuscript for important intellectual content: NR, MR, CS, and TO. Administrative, technical, or material support: NR, MR, and CS.

## Conflict of Interest Statement

TO and NR hold patent on AIM technology. None of the authors has any relevant financial disclosures.
